# Remediation of learners struggling with communication skills: a systematic review

**DOI:** 10.1186/s12909-020-02074-9

**Published:** 2020-07-09

**Authors:** Deema Al-Sheikhly, Linda Östlundh, Thurayya Arayssi

**Affiliations:** 1 Division of Continuing Professional Development, Weill Cornell Medicine – Qatar, Doha, Qatar; 2 National Medical Library, United Arab Emirates University, Al-Ain, UAE; 3 Medical Education and Continuing Professional Development, Weill Cornell Medicine – Qatar, Doha, Qatar

**Keywords:** Assessment, Communication skills, Remediation, Intervention, Systematic review, Healthcare practitioners, Learners

## Abstract

**Background:**

Communication skills is a core area of competency for healthcare practitioners. However, trainees deficient in those skills are not identified early enough to address the deficiency. Furthermore, faculty often struggle to identify effective remediation strategies for those who fail to meet expectations. We undertook a systematic review to determine which assessment methods are appropriate to identify learners that struggle with communication skills and the strategies used to remediate them.

**Methods:**

The literature was searched from January 1998 through to May 2019 using academic databases and grey literature. Trainees were defined as healthcare practitioners in undergraduate, graduate and continuing education. Characteristics of studies, assessment and intervention strategies and outcomes were synthesized qualitatively and summarized in tables.

**Results:**

From an initial 1636 records, 16 (1%) studies met the review criteria. Majority of the learners were medical students. A few studies (44%) included students from other disciplines, residents and physicians in practice. The remediation programs, in the studies, ranged from 1 week to 1 year. Around half of the studies focused solely on learners struggling with communication skills. The majority of studies used a format of a clinical OSCE to identify struggling learners. None of the studies had a single intervention strategy with the majority including an experiential component with feedback.

**Conclusions:**

A few studies collectively described the diagnosis, remediation intervention and the assessment of the outcomes of remediation of communication skills. For a remediation strategy to be successful it is important to ensure: (i) early identification and diagnosis, (ii) the development of an individualized plan and (iii) providing reassessment with feedback to the learner.

## Background

Interpersonal and communication skills are an integral element of quality patient care and are recognized as a core area of competency for medical students, residents, and practicing physicians [1–5]. Furthermore, effective communication and empathic relationships with healthcare practitioners are highly valued by patients and their families [6–10] with compromised clinical care and an overall lower satisfaction with care being associated with poor communication [11–13]. Hence formal training and assessment programs at the undergraduate, graduate and continuing education levels are needed [14–17]. Examples of these include Objective Structured Clinical Examination (OSCE) with a Standardized Patient (SP), that assesses clinical skills in a standardized setting. Although medical schools have a variety of methods to teach communication skills, there still remains a considerable gap. Not all communication curricula are based on a specific validated framework, nor incorporate a patient-centered communication approach, nor foster professional and personal growth. Additionally, the learners’ communication skills may not always be assessed directly and the quality of the program may not be evaluated [17].

In 1999, the Accreditation Council for Graduate Medical Education (ACGME) and the American Board of Medical Specialties (ABMS) stated that “*interpersonal and communication skills that result in effective information exchange and partnering with patients, their families, and professional associates*” is a core area of competency for residents and practicing physicians [1, 4]. Additionally in 2004, the National Board of Medical Examiners (NBME), the Federation of State Medical Boards (FSMB), and the Educational Commission for Foreign Medical Graduates (ECFMG) implemented the Step 2 Clinical Skills (CS) Examination [18]. One of three subcomponents of the exam is Communication and Interpersonal Skills, which requires medical students or graduates to “establish rapport with the patients, gather and provide information, help the patient make decisions and provide counseling when appropriate and in a professional manner” [18].

Despite the importance of communication skills to the training of future healthcare practitioners and the requirement to demonstrate competence in those skills at all levels of the medical continuum, faculty and residency program directors often struggle with identifying effective remediation strategies for those who fail to meet expectations [19–21]. This has mainly been due to the fact that remediation is a time consuming process that can be daunting and cumbersome [22] and that remediation of non-cognitive problems is more challenging than remediation of cognitive problems [20, 23].

The literature has shown that policies and guidelines for best practice are needed to improve the quality of the remediation process and to increase the confidence of educators in applying specific remediation strategies according to the learner’s skill deficit in all areas of competencies [23, 24]. A variety of remediation strategies have been utilized with most consisting of three steps: identification/diagnosis, remediation intervention, and re-assessment [25–27]. Hauer et al. proposed a four-step model which included: (i) initial assessment to identify deficiencies using multiple assessment tools, (ii) diagnosis and development of an individualized learning plan, (iii) deliberate practice, feedback, and reflection, and (iv) reassessment [23]. A structured seven-step approach of relationship-centered care, coaching and effective feedback was also found to be an effective model to successfully remediate learners in communication and interpersonal skills [28]. Some of the key steps included establishing a supportive learning environment, listening to the learner, encouraging reflective practice, developing a learning plan and documenting progress.

The literature on challenges in identifying and remediating learners struggling with communication skills are wide and varied [28]. Therefore, the goal of this systematic review was to determine the appropriate assessment tools used to identify learners struggling in communication skills, the strategies used to remediate them and to discuss the best practice recommendations proposed by the authors. In this study we defined remediation as “additional teaching above and beyond the standard curriculum, individualized to the learner who without the additional teaching would not achieve the necessary skills for the profession” [29].

To achieve this, our research question was:

How do you diagnose a trainee struggling with communication skills and what are the effective remediation strategies?

## Method

In this systematic review a PRISMA (Preferred Reporting Items for Systematic Reviews and Meta-Analysis) flow diagram was utilized for reporting the study selection.

### Data sources and search strategy

A comprehensive, search for literature was performed in the academic databases PubMed, MEDLINE (OVID), EMBASE (OVID), CINAHL (EBSCO), PsycInfo (OVID), Web of Science and Scopus and in sources of grey literature. Pre-searches to identify relevant search strategies, search terms and information sources were conducted in March–June, 2018, and the final search was carried out in June 2018. An update of the search in PubMed and Scopus were performed in May 2019 to ensure inclusion of the latest published studies on remediation in communication for healthcare practitioners before completing the manuscript.

PubMed was used to systematically develop a search string, which later was applied in the other databases. All selected keywords were searched both in the fields “Abstract” and “Article Title” (alternatively “Topic”) and in MeSH/Subject Headings/Thesaurus when available. No filters or limitations were applied to retrieve the largest number of result and to avoid excluding pre-indexed materials. Language, document type, and publication year restrictions were instead included in the exclusion criteria for the screening process. We defined trainees as healthcare practitioners in undergraduate, graduate and continuing education. For the purpose of this study we defined healthcare practitioners as individuals who may be involved in healthcare delivery (for example: physicians, nurses, dentists, physiotherapists and pharmacists). A full search log, including detailed search strings for all included information sources, results and notes is available in Appendix.

Searches for grey literature were conducted in ProQuest Dissertation and Thesis, Ethos, Open Grey and BASE, The New York Academy of Grey Literature Reports and in the library catalogues for British Library, Library of congress and WorldCat. Due to lack of advanced search features in many of the grey resources, broader search strings than the one used in the academic databases had to be applied. The grey search was updated in May 2019. A full search log can be found in Appendix.

All the references were uploaded into Covidence (Melbourne, Australia), systematic review software for blinded screening. Duplicate detection and removal were carried out using this software.

To complete the selection of relevant references for the review, a systematic hand screening of references lists in studies identified to be included in the systematic review was also carried out. Two additional studies were identified eligible for the systematic review.

### Study selection and title and abstract review

**Fig. 1:**
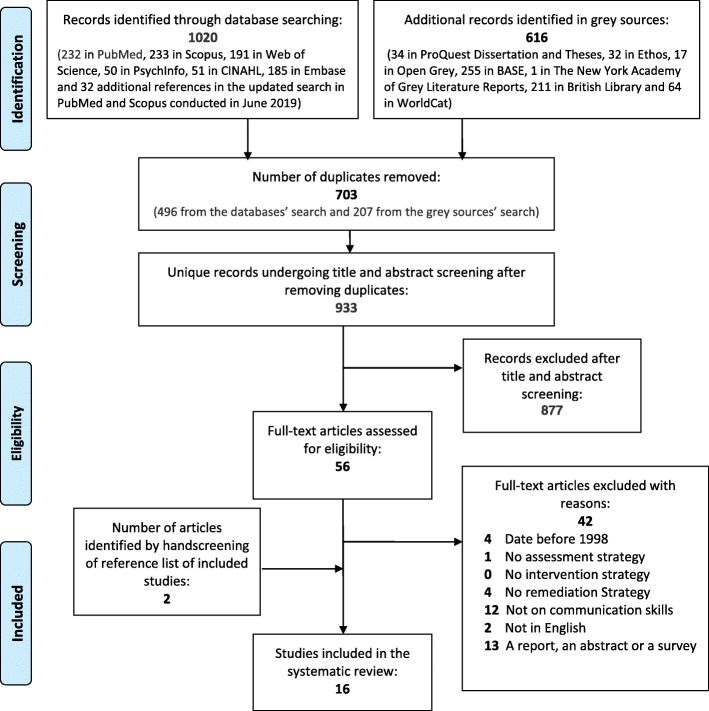
PRISMA flow diagram [30]

Articles were included if they were original research on remediation in the area of interpersonal and communication skills. Articles that were not written in the English language, systematic reviews, conference abstracts, proceedings, book chapters, comments, editorials or letters and publications prior to 1998 were excluded. We wanted to limit the review to primary studies following the implementation of the Accreditation Council for Graduate Medical Education’s (ACGME) outcome project where competencies for training, including communication skills, were defined and implemented. The search in academic databases and in grey sources yielded 1636 articles (Fig. 1). Based on the title and abstract, the two reviewers (DA and TA) screened the articles using Covidence and excluded articles that were clearly irrelevant. The screening in Covidence was blinded. In situations where it was difficult to determine eligibility based on the title and abstract review the article was included for full article review. The authors met regularly and all uncertainties were resolved by consensus. Only articles that described an assessment tool to identify struggling learners as well as an intervention methodology or remediation strategy were included. Articles with an assessment and remediation strategy but no clear outcome were also included.

### Data extraction

Data were entered into a structured extraction framework that we created. The framework included information on the article (Year, participant level, participant number, country of study), assessment tool, remediation strategy and intervention outcome. One author (DA) extracted the data.

The Medical Education Research Study Quality Instrument (MERSQI) used to evaluate the methodological quality of experimental, quasi-experimental, and observational studies in medical education was used [31]. The tool includes 10 items, reflecting 6 domains of study quality [study design, sampling, type of data (subjective or objective), validity, data analysis, and outcomes]. The potential range of a MERSQI score is 5–18. Each study was scored at the highest possible level.

We used the Kirkpatrick’s four-level model for evaluation of educational interventions to classify the outcomes of the studies that met our selection criteria [32]:

- Level 1: Participant reaction assessed (learner satisfaction).
- Level 2: Participant learning assessed (changes in knowledge and skills).
- Level 3: Participant behavioral change assessed (application in practice).
- Level 4: Results (changes in institutional practice and patient outcome).

### Data synthesis

Characteristics of studies, assessment and intervention strategies and outcomes were synthesized qualitatively and summarized in tables. Articles that described similar assessment tools or similar interventions were grouped to facilitate the analysis. We used the consensus mean MERSQI scores with standard deviations to describe the overall quality of included studies. Meta-analysis was not possible, given the heterogeneity of assessment tools, interventions and outcome measurements.

## Results

### Characteristics of eligible studies

From the 1636 records found, 1020 were identified through electronic database searching and 616 were identified through other resources such as databases for grey literature and by systematically reviewing citations in studies selected to be included in the review. After de-duplication, a total of 933 references were identified for a first review. The total number of articles that were eligible for inclusion through review of titles, abstracts and full texts was 16 (Fig. 1). Table 1 summarizes information on the country of the study, the level of struggling learner, number of remediated learners, assessment tools, interventions and outcomes. Struggling learners in the studies were predominantly students (*n* = 11, 69%), followed by residents (*n* = 4, 25%), and one study included family physicians and specialists (n = 1, 6%). Only one study included pharmacy students (6%), 14 included learners in medicine (88%) and one included both nursing and medical students (6%). Eight studies were conducted in the US, three in the UK, two in Canada, one in Belgium, one in Australia and one in Korea. Only seven studies focused solely on learners struggling with communication skills [33–39] while the remaining studies addressed multiple deficiencies.

**Table 1:** Summary of characteristics of eligible studies

First Author and year	Number & Struggling learner Characteristic & Country	MERSQI Score	Description of Assessment for identifying struggling learner	Intervention	Outcomes (Kirkpatrick level of evaluation)
Bodenberg 2015 ^(44)^	8 Pharmacy Student US	8	• Midpoint evaluation and concern cards to alert the Director of Experiential Education of potential behavioral or learning issues. • The faculty developed a section in the student evaluation form in which the preceptor can suggest longitudinal monitoring or remediation needed for the student. • 9% had a communications skills deficiency.	The Director of Experiential Education creates a performance improvement plan. This individualized plan takes into account input from the student, preceptor, and the Director of Experiential Education. A list of communication tools includes: • Direct observations of the student during case presentations, • Counseling, • Drug information, • Topic presentations, and • An oral “End of Block” exam.	The on-time graduation using the developed remediation plan was seven of eight students (88%), and the overall graduation success rate was eight of eight students (100%). ***(Level 2)***
Chang 2008 ^(40)^	23 Medical Student US	5.5	The clinical performance examination (CPX) is an eight-station, high-stakes examination in which SPs assess early Year 4 medical students’ competence in clinical and communication skills. Students scoring ≥2 standard deviations (SD) below the class average in one or both skill domains (history taking and physical examination [H & P] or patient–doctor interaction [PDI]) are required to remediate.	Developed a four-step CPX remedial training program that incorporates diagnosis of learner problems, individual reflection, faculty feedback and supervised practice. **Step 1** consists of an individual review in which: • Students watch their own videotaped examinations, • Reflect on their performances, and • Develop personal learning goals. **Step 2,** the faculty remediation director: • Watches the videotape independently and • Generates a ‘learning prescription’, **Step 3** involves a one-to-one video review guided by the learning prescription and the student’s self-identified goals with a designated faculty member. **In Step 4,** students attend one or both evening workshops in H & P or PDI, depending on their area of need. Workshops consist of: an introductory 30-min didactic session addressing basic concepts; case-based skills exercises in which subsets of 2–3 students rotate between examination rooms with a faculty facilitator and an SP, and a whole-group concluding session to review learning goals.	Students who require remedial training in clinical and communication skills appreciate individualized feedback and skills sessions with preceptors and SPs. According to personal communication, all students in the program succeeded. ***(Level 1)***
Deveugele 2005^(32)^	Medical Student Belgium	7.5	• Following a longitudinal communication curriculum, students assessed every year using an OSCE with SP with specific objectives. • Two examiners rate the student and come to a final mark after discussion.	Three different remediation strategies depending on year level: • During the 2nd year of the Bachelor degree, remedial teaching consists of one up to three extra training sessions in a small group. • During the 1st year of the Master education, every student who failed is invited to exercise during 1 h with a simulated patient. The student can formulate his/her own learning objectives. The whole session is videotaped and the taped is reviewed by one of the trainers in order to give feedback to the student. The student can rehearse this three times. • During the 2nd year of the Master degree, the student exercises with a simulated patient in the presence of one of the trainers and gets immediate feedback.	No outcome stated
Dowell 2006^(33)^	28 Medical Student UK	8	• OSCE to assess communication skills “Consultation skills” as a screening tool. • Three 4-min OSCE to assess aspects of communication skills. • Scoring was done using the SEGUE framework by trained tutors.	• Attend a week of additional training. • Interactive teaching contained many opportunities to develop, videotape, review and practice both basic and more complex consultation skills. • At the end, a separate four-station OSCE was used to assess skills using another three four-minute consultation skills stations plus an additional ten-minute assessment that allowed students to complete a basic consultation. • This was videotaped to enhance feedback and reliability.	Students succeeded and progressed to next year. ***(Level 2)***
Goulet 2005^(41)^	220 Family Physician and 85 Specialist Consultant Canada	9	Physicians with clinical performance problems identified through: • Professional Inspection Committee (PIC), • Complaints forwarded to the inquiry division, or • Processes initiated by physicians who would like to re-orient their careers or come back to practice after a period of inactivity of over four years. Every assessment process is conducted using a: • Standard grid listing the criteria of quality of care, • Chart keeping, and • Office practices. Sometimes a more in-depth evaluation is conducted: • An evaluation of specialists’ clinical performance by a peer. • The structured oral interview (SOI). Six objective structured clinical examination (OSCE) stations were added to the SOI. (includes knowledge, Physical exam, Doctor-patient relationship)	To improve Physician practice, the Professional Inspection Committee (PIC) may recommend that: 1. The physician participates in specific CME activities. 2. The CMQ admin committee imposes a remedial retraining. Interventions include: • Clinical training programs, • Tutorials, • Focused readings, and • Various courses or workshops developed and organized by the CMQ in association with the medical schools in Quebec or the Quebec College of Family Physicians.	• 70% of the retraining activities led to attainment of the training objectives; • 15% led to partly attained objectives, • 13% failed to lead to attainment of objectives, and • 2% involved missing data or withdrawal. The 70% resumed their practices. ***(Level 2)***
Guevara 2011^(45)^	16 IM, Diagn Rad, Ob/Gyn, ER, Gen Surg, Peds Residents US	8	• Monthly evaluation from Faculty, peers and students. • 360-degree evaluation, which includes input from nurses and case managers. • Evaluations reviewed by Internal Residency Review Committee (IRRC). • The IRRC charges the PD or Chief Resident to counsel the resident and monitor their progress. If no improvement formal IEP may be required.	• Individualized Education Plans (IEP) that includes a listing of competencies, intervention designed to address, reassessment with objective metrics and milestones for completion of IEP. • Resident input is solicited to refine the IEP and to identify a faculty mentor. • Interventions include, remedial tutorials, frequent meetings with mentor, speech therapy, counseling	12/16 successfully graduated from the IEP program. ***(Level 2)***
Lin 2001^(34)^	1 Medical Student US	8	• Clinical preceptors assessment and end-of-year clinical practice exam. • OSCE/SP encounters in communication Skills.	A year-long intensive remedial curriculum in communication skills. Includes: • Pairing with a clinical preceptor for intensive skills training, including a weekly preceptor clinic, • Structured readings, • SP exercises, • Communications workshops, and • End-of-year standardized clinical evaluations.	Improvement in communication skills. Additionally the student unexpectedly wrote a 12-page guide to interview skills for his preclinical colleagues. ***(Level 2)***
Malau-Aduli 2013^(46)^	18 Medical Students (4th and 5th year) Australia	11	Students were identified for remediation due to the following: • Failure in examinations or repeating the year. • Workplace-based assessments.	Remediation program developed based on socio-cognitive Self-Efficacy beliefs to improve academic and clinical performance. • A multi-dimensional ten-week support programbased on individual assessment offered primarily as a group learning experience. • Individual counseling to provide psychological support. The program consisted of the following elements: • Presentation Skills workshops • OSCE Practice (4-station) with Clinical Teachers • OSCE Practice with other Students • Bedside Teaching	Performance on all measures improved after the remediation program with statistically significant improvements on management plan (MP), diagnostic skills (DS), communication skills (CS) and number of stations passed (NSP). All 18 participants in the remediation program were successful in their end of 4th year summative OSCE assessments. In their 5th year (eight of them) passed all their examinations without any support or intervention. ***(Level 2)***
Myung 2013^(42)^	23 Medical Students Korea	10	Clinical performance examination (CPX) (8 stations with SP encounters using a checklist).	A six-week remediation program (3 weeks Internal Medicine classes and 3 weeks Family Medicine classes). Includes: • 1:1 tutoring sessions • Re-examination • Feedback from SP Designed with 3 parts: Diagnosis, learning activities, and re-examination.	• Students’ scores on the CPX exam improved. ***(Level 2)***
Rowland 2012^(47)^	225 Surgery Residents US	9.5	• Identified by Directors: (Mock Oral Exams, Case Presentations, Journal Clubs, Mortality & Morbidity conferences, outpatient clinics, and hospital and operating room settings). • Failed Surgery Certifying Exam. • Rowland Communication Skills Inventory used to identify candidates with severe communication problems that might need further assistance with their communication skills before entering the course.	5-Day Oral Examination Course: • Didactics, • Mini oral examinations in suites, • Individual assessments, • Small-group exercises, • Formal mock oral examinations, • Individual debriefing sessions with a general surgeon and a behavioral scientist that summarized individual improvement, communication competency, strengths and weaknesses on the formal mock oral examination, and a remediation plan for future improvement. Resident received a personal digital video of their formal mock oral for review and self-critique and individual remediation plan. In 2007, course shortened to 3 Days.	Primary outcome measure is the successful completion of the Certifying Exam. 218 residents followed their remediation plan and successfully Passed the Surgery CE on first attempt ***(Level 2)***
Ryan 2010^(36)^	64 Nursing 46 Medical Students UK	7	A screening program in communication and consultation skills (CCS) using: • Trained Standardized Patient Educators (SPEs) • A previously validated global rating scale for CCS. Almost three quarters of medical students (33/46; 72%) and 81% of nursing students (56/64) passed the CCS assessment in both communication and attitudes categories.	One-on-one CCS training.	***(Level 2)***
Saxena 2009^(24)^	Medical Students US	11	Comprehensive Assessment Test (Cross-disciplinary exam with SPs). • Reviewing exam scores (96%) • Reviewing video of failing student exam (57%) • Meeting with failing stud (49%)	• Precepted video review • Preceptorship • Independent Study (Web-Based module, reading) • Stud independently reviews exam recording • Practice with SP • Skills workshops, seminars or group discussions	• Study measured confidence and not outcomes of remediation. ***(Level 2)***
Sperry 2010^(37)^	3 Medical Students (4th yr) US	11	Clinical performance examination (CPX) using SP. • Evaluation includes Medical History, physical exam, communication and relationship issues, diagnosis and management.	Individualized Doctor-Patient Communications and Psychosocial Interviewing remediation curriculum addresses communication skills deficits: • 2-week Didactic and experiential components including role-play, videos, personal reflections, performing interviews, history taking and Physical exam with patients presenting to clinic. • Observed Live and provided feedback by family physicians. • Written test assess knowledge of communication strategies before and after remediation. • Patients completed a satisfaction survey.	• No difference in written test scores. • Patient satisfaction indicated positive qualities with no difference before and after. • Preceptors’ evaluation of students indicated an improvement in CS. • All passed CPX and one passed a re-sit of USMLE-CS ***(Level 2)***
Torbeck 2009^(20)^	Surgery Residents US	6	• Assessment tools used were not addressed.	Program director devise individual remediation plans and monitor progress. Most programs use primarily 3 methods for remediating residents: • To increase direct observation of the resident by the attending in the clinic/operating room/wards, • To have the resident undergo psychological counseling, and • To have the resident attend organized professionalism or communication workshops/seminars. Among the other methods reported: • 360° evaluations, • Specific counseling with the PD, • Reviewing How to Win Friends & Influence People on a weekly basis with the program director, • Have residents present frequently, • Sending a resident to an English tutor, • Recommending a speech/communication coach, and • Counseling for problems related to hostile relationships/interactions.	***(Level 1)***
Wiskin 2013^(38)^	1 Medical Student UK	8	• Clinical OSCE	• One-on-One coaching • Individual and group teaching, • Individual support and remedial teaching, • Workshop program, • OSCE course.	No outcome stated
Zbieranowski 2013^(39)^	100 Medical Resident Canada	8	• Identified by Failure to meet criteria of CanMEDS roles. • Board of Examiners for Postgraduate Programs (BOE-PG) objectively review cases of postgraduate students in academic difficulty and determine appropriate course of action, which could include: Remediation, probation, or dismissal. • *49% had weakness as Communicator CanMed Role.*	CanMEDS Roles are used as the organizational framework for the individual formal remediation plans developed by the residency program director.	78% Completed Residency Education. ***(Level 2)***

### Quality of studies

The mean consensus MERSQI score was 10.5 (range 5.5–11), with a standard deviation of 1.67 and a median score of 8.5, indicating that the overall study quality was not high. Total consensus MERSQI scores for each paper are shown in Table 2. Mean domain scores were highest for type of data (2.63), data analysis (2.19) and sampling (2.06); they were lowest for validity evidence (1.17) and study design (1.13). Most of the studies (81.3%) were single group cross-sectional or single group post-test only. One study was a retrospective review of records [40], two studies were surveys of medical schools [24, 39] and one study was a survey of surgery residency programs [20].

**Table 2:** The MERSQI^a^ domain and item scores for the 16 selected studies that meet the review criteria

Domain	Item	Studies N (%)	Score		Mean (SD)	
			Item	Maximum Domain	Item	Domain
***Study Design***				3	1.13 (0.29)	1.13 (0.29)
1.Study Design						
	Single group cross-sectional or single group post-test only	13 (81.3)	1			
	Single group pre and post-test	2 (12.5)	1.5			
	Non-randomized, 2 group	1 (6.3)	2			
	Randomized controlled experiment		3			
***Sampling***						
2. Institutions				3	0.81 (0.48)	2.06 (0.92)
	Single institution	11 (68.8)	0.5			
	Two institutions		1			
	More than 2 institutions	5 (31.2)	1.5			
3. Response Rate					1.25 (0.50)	
	Not applicable	10 (62.5)	n/a			
	Response rate < 50% or not reported	1 (6.3)	0.5			
	Response rate 50–74%		1			
	Response rate ≥ 75%	3 (18.8)	1.5			
***Type of Data***						
4. Type of Data				3	2.69 (0.75)	2.69 (0.75)
	Assessment by study subject	2 (12.5)	1			
	Objective measurement	14 (87.5)	3			
***Validity of Evaluation Instruments’ Scores***						
	Not applicable	9 (56.3)	n/a			
5. Internal Structure				3	0.50 (0.55)	1.17 (1.33)
	Not reported	3 (18.8)	0			
	Reported	3 (18.8)	1			
6. Content						
	Not reported	3 (18.8)	0		0.50 (0.55)	
	Reported	3 (18.8)	1			
7. Relationships to other variables						
	Not reported	5 (31.2)	0		0.17 (0.41)	
	Reported	1 (6.3)	1			
***Data Analysis***						
8. Appropriateness of analysis				3	1.0 (0.0)	2.19 (0.40)
	Data analysis inappropriate for study design or type of data		0			
	Data analysis appropriate for study design and type of data	16 (100)	1			
9. Sophistication of analysis					1.19 (0.40)	
	Descriptive analysis only	13 (81.2)	1			
	Beyond descriptive analysis	3 (18.8)	2			
***Outcome***						
10. Outcome				3	1.37 (0.23)	1.37 (0.23)
	Satisfaction, attitudes, perceptions, opinions, general facts	4 (26.7)	1			
	Knowledge, skills	11 (68.8)	1.5			
	Behaviors		2			
	Patient/health care outcome		3			
**TOTAL**				18		10.6 (1.65)

^a^Medical Education Research Study Quality Instrument

### Assessment methods used to diagnose struggling trainees

Table 1 provides details of the assessment tools that were used in the studies to diagnose trainees struggling with communication skills and Table 3 provides a summary of the overall assessment methods used in the studies reviewed. Most studies (*n* = 10, 62.5%) used a format of a clinical OSCE, a tool to assess clinical skills in a controlled setting, to identify struggling learners [33–35, 37–39, 41–44], four (25%) used a 360-degree or peer evaluation tool [42, 45–47], one study did not address assessment methods used [21] and another identified struggling learners by their failure to meet criteria in one or more CanMed Roles but did not expand on what tools were used to achieve this [40].

**Table 3:** Summary of assessment methods and remediation strategies

Assessment Methods	Remediation Strategies and Methods
• OSCE/SP using a global rating scale • OSCE/SP using a standardized checklist • Structured oral interview with an OSCE evaluation • Direct observation of clinical encounters • Direct observation of role-plays • MiniCEX • Monthly Evaluation • 360° evaluation • Mid-point evaluation • Workplace-based assessments • Patient surveys • Oral certifying Exams • Rowland Communication Inventory	**Didactics** • Tutorials on communication related topics • Workshops on presentation skills, doctor-patient relationships and other communication topics • Viewing triggers tapes • Large group sessions **Observations** • OSCE Practice with SP, Clinical Faculty or other peers • Direct observation of clinical encounters using global rating scale or checklist • Direct observation of role-play using global rating scale or checklist • Observation of faculty interacting with patients (role-modeling) followed by discussion • Small group practice sessions (Clinical interviews) • Performing interviews with patients **Reflection and Assessment** • One-to-one review • Video Reviews followed by self-assessment and feedback **Other** • Coaching and mentoring • Written tests on knowledge of communication strategies and behavioral issues • Participation in CME activities

(Table 3: Summary of Assessment Methods and Remediation Strategies) – Insert near here.

### Remediation interventions and outcomes

The studies included a wide range of intervention strategies such as one-on-one coaching/mentoring, tutorials, individual and group work, focus reading, SP exercises, role-play, videotape review, and counseling (Table 1). None of the studies had a single intervention strategy with the majority including an experiential component with feedback. Half of the studies (*n* = 8) developed a remediation course or program [33–36, 38, 41, 43, 47] with the duration of the intervention ranging from as short as a weeklong course [34] to a longitudinal one-year program [35].

Five studies (35.3%) had a program director or a committee to devise an individualized remediation plan that included input from learners [20, 37, 40, 45, 46] and one of those studies used the CanMed Roles as a framework for the development of the plan [40]. In one Canadian study on improving physicians in practice a variety of intervention strategies were used. However, the strategies that were used to remediate deficiencies in communication skills were not specified [42]. A study that surveyed how medical schools in the UK support students struggling with communication skills found that some schools had a structured remediation program that included coaching, one-on-one encounters and simulated patient intervention. However, most schools used an ad hoc approach [39]. Only one study on medical students in Australia developed a remediation program based on a learning theory [47].

Based on Kirkpatrick’s model of educational outcomes [32], three (18.8%) of the studies assessed reaction, which was based on learner satisfaction and appraisal of the program [20, 33, 41, 44]. Eleven (68.8%) assessed learning, which included changes in knowledge and skills [34, 35, 36, 37, 38, 40, 42, 43, 45–47].

## Discussion

This systematic review on the remediation of deficiencies of interpersonal and communication skills of healthcare practitioners across the continuum yielded very few studies that described the diagnosis, remediation, intervention and the assessment of the outcomes of remediation. Furthermore, the studies that we identified were small scaled (range: *n* = 1 to *n* = 225) and of single-institutions. They utilized a variety of assessment methods to diagnose the specific problems the learners were struggling with including evaluations, clinical performance exams, OSCEs with SPs, direct observations, oral certifying exams and global rating scales. This is similar to the recommendations from the Kalamazoo II report that outlined specific assessment methods to evaluate communication skills [48]. Those included (i) direct observations with real patients, (ii) ratings of simulated encounters with real patients, (iii) ratings of video or audiotaped interactions, (iv) patient surveys and (v) examinations of knowledge, skills or attitude.

In our study, OSCE with SP was the most widely used method for assessing the learners with the majority utilizing a standardized or validated checklist. According to the literature OSCE with SPs is considered the “gold standard” tool for clinical assessment [49] as it can be designed to examine skills and ability at the “Show how” level of Miller’s triangle [50]. The checklist is thought to be the most frequently used assessment tool of communication behavior as it provides clearer behavioral definitions that may improve reliability [51]. In one of the studies, the students rated practicing with SPs, receiving feedback, from SPs and faculty, in real time and observing others in small groups to be the most beneficial components of the program that helped them improve in their communication skills [41]. This was also observed in other studies that included OSCEs with opportunities for video review and feedback as part of the remediation intervention [34, 43].

Deficiencies in non-cognitive skills are the most challenging to remediate [52]. Therefore, it was not surprising that our systematic review identified a lack of standardized remediation programs for learners struggling with communication skills. However, we identified common themes for remediation strategies, which included the use of clinical practice with an SP, a clinical faculty or another peer, reflective practice, role-play, video review and structured feedback. Having institutional policies and guidelines for remediation, a faculty development as well as a mentoring program, using learning contracts and documentation of every aspect of the remediation process are important components that support the success of the remediation plan. The challenge in the systematic review was that there were no clear outcomes specified in most of the remediation programs other than the learners progressing to the next year of their education program, passing a certifying exam or graduating.

Moreover the results from this systematic review confirm what was previously published in that there is a deficiency of outcomes-based research on strategies for remediation [23] and a lack of standardized remediation programs [53, 54]. Three steps that lead to successful remediation were identified in our study that are similar to those identified in previous studies: (i) early identification and diagnosis, (ii) developing an individualized remediation plan and (iii) re-assessment and feedback [22, 26, 53, 55, 56].

Our study further confirmed what was previously described in that remediation interventions lack theoretical foundation and clinical teachers struggle with using a structured process framed by appropriate theory to generate a specific educational diagnosis of learners’ difficulties [55]. The majority of the studies we reviewed did not utilize theory to develop their remediation plan. Only one study used theory (socio-cognitive self-efficacy beliefs) to develop the remediation program [47] and the authors noted that participants benefited from enhanced self-efficacy beliefs. Adult learning theory is thought to have a direct impact on remediation as the relevance of what is taught as well as self-direction are important since each learner has their own approach based on their life experiences [57]. Kolb’s experiential learning cycle [58] has been successfully used to develop remedial courses for surgical residents struggling with the surgery-qualifying exam [57] and for residents deficient in communication skills, namely clinical interviewing skills [59]. Kolb’s experiential cycle has multiple intercalations with many educational theories. Therefore designing an experiential remedial program using educational activities that mirror principles of educational theories would be beneficial [59]. Such activities would target various increasing levels of cognitive development [60], provide supportive corrective feedback [61] and reflective practice [62]. In both of the above studies, the strategies used included learning contracts, structured reflection, reviewing videos or reading material and role modeling. Although the majority of authors in the studies we reviewed do not mention the use of learning theories to develop their remediation plans they have unknowingly done so. Most of the remediation strategies used included a clinical experience (concrete experience), an observation and reflection on that experience for example reviewing the video recordings of encounters (reflective observation), conceptualizing and learning from that experience as well as learning new techniques for example through didactics and role-plays (abstract conceptualization) and finally deliberate practice to apply what was learned (active experimentation) and immediate feedback. These are the main components of Kolb’s experiential cycle [58].

In our study, several remediation plans included reflection by the learners following their OSCEs, role-plays or clinical encounters with patients. Reflection before, during and after an action is foundational to self-directed learning and is necessary to promote learning. Using Schon’s model [62] of the reflective practitioner provides those learners with a framework for choosing an effective action in a complex situation. It is important for the learners to be able to develop the capacity to derive lessons from a concrete clinical experience [58]. Such experiences help them refine their skills and apply their learning to subsequent encounters. By actively reflecting on what they do and do not understand, they can enhance their own learning from the concrete experience, which in turn may facilitate the potential transformative impact [63].

### Strengths and limitations of the review

The major strengths of this study lie in the search process itself, which was very comprehensive and included a wide range of academic databases as well as grey literature. Additionally, we did not limit the study to one group of learners and included all healthcare practitioners across the continuum. The study however has several limitations. First, the data extraction was performed by a single author, and did not include conference abstracts, proceedings, book chapters or articles that only described an assessment tool to identify struggling learners or an intervention methodology or remediation strategy. Second based on the MERSQI score, the quality of the studies included were not high and that is a limitation of the work conducted in the area of study. Furthermore, the studies included in this systematic review were heterogeneous and hence we were not able to perform a meta-analysis. There was not enough data to indicate whether institutions that remediate trainees struggling with communication skills assess their own communication training programs to identify any deficits that could be addressed. Additionally, it was not possible to investigate the structural differences of the 16 studies identified due to the variation in the type of information provided.

### Implications for practice and future work

Despite these limitations, we can make some recommendations based on our observations from the studies reviewed. Having regular evaluation and feedback methods in place may facilitate the identification of deficiencies early to avoid serious learning problems later on [64]. For a remediation strategy to be successful it is important to ensure early identification and diagnosis, the development of an individualized plan and reassessment with feedback. The most effective methods for teaching and evaluating interpersonal and communication skills involve multiple methods of assessment [3]. Therefore, we would recommend using multiple methods that would include direct observations (with patients, SPs or via video review] using a checklist or global rating scale, 360-degree evaluations, patient surveys, case discussions, role-plays or written examinations of knowledge, skills or attitude. Following the diagnosis of the problem the next steps would include discussions with the learner in order to develop an individualized remediation plan, having a learning contract, setting clear goals and objectives, a reasonable timeline, assigning a mentor, ongoing monitoring, deliberate practice, re-evaluation and feedback.

## Conclusion

This study supports the need for more rigorous outcomes-based research, using control or comparison groups, for the diagnosis and remediation of healthcare practitioners struggling with interpersonal and communication skills across the continuum. It is important to consider the following practice points: (i) deficiencies in non-cognitive skills are challenging to remediate, (ii) a major challenge is whether faculty know how to identify the deficiency and what strategies to use to remediate, (iii) a variety of assessment tools need to be used to evaluate communication skills and (iv) early identification and diagnosis, creating an individualized plan and reassessment with feedback are key to successful remediation.

## Data Availability

All data generated or analysed during this study are included in this published article.

## Appendix

### Literature search

**Table 4:** Academic databases

Source and search date	Search String	Result	Notes
**PubMed** **Search Date:** 2018-06-08 **Coverage:** 1809-	((“psychiatrists”[Title/Abstract] OR “psychiatrist”[Title/Abstract] OR “premedical education”[Title/Abstract] OR “dental education”[Title/Abstract] OR “dental educations” [Title/Abstract] OR “pharmaceutical education”[Title/Abstract] OR “pharmaceutical education” [Title/Abstract] OR “pharmaceutical school” [Title/Abstract] OR “pharmaceutical schools” [Title/Abstract] OR “pharmaceutical student” [Title/Abstract] OR “pharmaceutical students” [Title/Abstract] OR “pharmacy education”[Title/Abstract] OR “pharmacy educations”[Title/Abstract] OR “nursing school”[Title/Abstract] OR “nursing schools”[Title/Abstract] OR “dental school”[Title/Abstract] OR “dental schools”[Title/Abstract] OR (“pharmacy school”[Title/Abstract] OR “pharmacy schools”[Title/Abstract] OR physiotherapist [Title/Abstract] OR physiotherapists [Title/Abstract] OR “physiotherapy education”[Title/Abstract] OR “physiotherapy students”[Title/Abstract] OR “physiotherapy student”[Title/Abstract] OR “physiotherapy school”[Title/Abstract] OR “physiotherapy schools”[Title/Abstract] OR “physical therapy faculty”[Title/Abstract] OR “physiotherapy faculty”[Title/Abstract] OR “physical therapy student”[Title/Abstract] OR “physical therapy students”[Title/Abstract] OR “physical therapy education”[Title/Abstract] OR “physical therapy educations”[Title/Abstract] OR “physical therapy school”[Title/Abstract] OR “physical therapy schools”[Title/Abstract] OR “Schools, Dental”[Mesh] OR “Schools, Nursing”[Mesh] OR “Schools, Pharmacy”[Mesh] OR “Education, Pharmacy”[Mesh] .OR “Education, Premedical”[Mesh]/ OR “Education, Dental”[Mesh] OR “Education, Nursing”[Mesh] OR “medical curricula”[Title/Abstract] OR “medical curriculum”[Title/Abstract] OR “medical faculty”[Title/Abstract] OR “medical learners”[Title/Abstract] OR interns [Title/Abstract] OR intern [Title/Abstract] OR internship [Title/Abstract] OR internships [Title/Abstract] OR “Education, Medical”[Mesh] OR “Internship and Residency”[Mesh] OR “Clinical Clerkship”[Mesh] OR “Students, Medical”[Mesh] OR “medical postgraduates”[Title/Abstract] OR “medical postgraduate”[Title/Abstract] OR “medical graduate”[Title/Abstract] OR “medical graduates”[Title/Abstract] OR “medical undergraduate”[Title/Abstract] OR “medical undergraduates”[Title/Abstract] OR “medical student”[Title/Abstract] OR “medical students”[Title/Abstract] OR “medical education”[Title/Abstract] OR “medical educations”[Title/Abstract] OR clerkship*[Title/Abstract] OR resident [Title/Abstract] OR residents [Title/Abstract] OR residency [Title/Abstract] OR “teaching round”[Title/Abstract] OR “teaching rounds”[Title/Abstract] OR “Health Personnel”[Mesh] OR “medical school”[Title/Abstract] OR “medical schools”[Title/Abstract] OR “medical college”[Title/Abstract] OR “medical universities”[Title/Abstract] OR “medical university”[Title/Abstract] OR “Schools, Medical”[Mesh] OR “physical therapists” [Title/Abstract] OR “physical therapist” [Title/Abstract] OR physician*[Title/Abstract] OR pharmacist*[Title/Abstract] OR nurse*[Title/Abstract] OR “medical staff”[Title/Abstract] OR “health educators”[Title/Abstract] OR “nursing faculty”[Title/Abstract] OR “dental faculty”[Title/Abstract] OR dentist*[Title/Abstract] OR “pharmacy faculty”[Title/Abstract] OR “health personnel”[Title/Abstract] OR “allied health personnel”[Title/Abstract] OR “Faculty, Nursing”[Mesh] OR “Faculty, Medical”[Mesh] OR “Faculty, Dental”[Mesh] OR “Students, Nursing”[Mesh] OR “Students, Pharmacy”[Mesh] OR “Students, Dental”[Mesh] OR “Students, Premedical”[Mesh] OR “premedical student”[Title/Abstract] OR “premedical students”[Title/Abstract] OR “pharmacy students”[Title/Abstract] OR “pharmacy student”[Title/Abstract] OR “dental student”[Title/Abstract] OR “dental students”[Title/Abstract] OR “nursing students”[Title/Abstract] OR “Nursing education”[Title/Abstract] OR “Nursing educations”[Title/Abstract] OR “nursing student”[Title/Abstract] OR “pharmacy graduates”[Title/Abstract] OR “pharmacy undergraduates”[Title/Abstract] OR “pharmacy graduate”[Title/Abstract] OR “pharmacy undergraduate”[Title/Abstract] OR “pharmacy postgraduates”[Title/Abstract] OR “pharmacy postgraduate”[Title/Abstract] OR “nursing graduates”[Title/Abstract] OR “nursing graduate”[Title/Abstract] OR “nursing undergraduates”[Title/Abstract] OR “nursing undergraduates”[Title/Abstract] OR “dental graduates”[Title/Abstract] OR “dental graduate”[Title/Abstract] OR “dental undergraduates”[Title/Abstract] OR “dental undergraduate”[Title/Abstract] OR “dental postgraduates”[Title/Abstract] OR “dental postgraduate”[Title/Abstract]) AND (“Remedial Teaching”[Mesh] OR Remediation*[Title/Abstract] OR remedial* [Title/Abstract]) AND (“Teach-Back Communication”[Mesh] OR “Health Communication”[Mesh] OR “Interdisciplinary Communication”[Mesh] OR “Persuasive Communication”[Mesh] OR “Nonverbal Communication”[Mesh] OR “Communication Barriers”[Mesh] OR “communication”[Mesh] OR communicat*[Title/Abstract] OR “interpersonal relation”[Title/Abstract] OR “interpersonal relationships” [Title/Abstract] OR “interpersonal relationship” [Title/Abstract] OR “interpersonal relations”[Title/Abstract] OR “interpersonal Interaction” [Title/Abstract] OR “interpersonal Interactions” [Title/Abstract] OR “Interpersonal Relations”[Mesh]))	**232**	All terms searched in field “Title/Abstract” and in “MeSH” when available. Filter for publication year applied
**Scopus** **Search Date:** 2018-06-08 **Coverage:** 1960-	(TITLE-ABS-KEY (“psychiatrists” OR “psychiatrist” OR “premedical education” OR “dental education” OR “dental educations” OR “pharmaceutical education” OR “pharmaceutical education” OR “pharmaceutical school” OR “pharmaceutical schools” OR “pharmaceutical student” OR “pharmaceutical students” OR “pharmacy educations” OR “nursing school” OR “nursing schools” OR “dental school” OR “dental schools” OR “pharmacy school” OR “pharmacy schools” OR physiotherapist OR physiotherapists OR “physiotherapy education” OR “physiotherapy students” OR “physiotherapy student” OR “physiotherapy school” OR “physiotherapy schools” OR “physical therapy faculty” OR “physiotherapy faculty” OR “physical therapy student” OR “physical therapy students” OR “physical therapy education” OR “physical therapy educations” OR “physical therapy school” OR “physical therapy schools” OR “medical curricula” OR “medical curriculum” OR “medical faculty” OR “medical learners” OR interns OR intern OR internship OR internships OR “medical postgraduates” OR “medical postgraduate” OR “medical graduate” OR “medical graduates” OR “medical undergraduate” OR “medical undergraduates” OR “medical student” OR “medical students” OR “medical education” OR “medical educations” OR clerkship* OR residents OR residency OR “teaching round” OR “teaching rounds” OR “health Personnel” OR “medical school” OR “medical schools” OR “Medical college” OR “medical universities” OR “medical university” OR “physical therapists” OR “physical therapist” OR physician* OR pharmacist* OR nurse* OR “medical staff” OR “health educators” OR “nursing faculty” OR “dental faculty” OR dentist* OR “pharmacy faculty” OR “health personnel” OR “allied health personnel” OR “premedical student” OR “premedical students” OR “pharmacy students” OR “pharmacy student” OR “dental student” OR “dental students” OR “nursing students” OR “nursing education” OR “nursing educations” OR “nursing student” OR “pharmacy graduates” OR “pharmacy undergraduates” OR “pharmacy graduate” OR “pharmacy undergraduate” OR “pharmacy postgraduates” OR “pharmacy postgraduate” OR “nursing graduates” OR “nursing graduate” OR “nursing undergraduates” OR “nursing undergraduates” OR “dental graduates” OR “dental graduate” OR “dental undergraduates” OR “dental undergraduate” OR “dental postgraduates” OR “dental postgraduate”) AND (TITLE-ABS-KEY (communicat* OR “interpersonal relation” OR “interpersonal relationships” OR “interpersonal relationship” OR “interpersonal relations” OR “interpersonal interaction” OR “interpersonal interactions”) AND (TITLE-ABS-KEY (remediation* OR remedial*))	**233**	All selected search terms searched in the fields for “Title”, Abstract” and “Keywords”, here marked with “TI-ABS-KEY.” No thesaurus available. No filters or limitations applied
**Web of Science** **Search Date:** 2018-06-08 **Coverage:** 1864-	((“Psychiatrists” OR “Psychiatrist” OR “Premedical education” OR “Dental education” OR “Dental educations” OR “Pharmacy education” OR “Pharmacy educations” OR “pharmaceutical education” OR “pharmaceutical education” OR “pharmaceutical school” OR “pharmaceutical schools” OR “pharmaceutical student” OR “pharmaceutical students” OR “Nursing school” OR “Nursing schools” OR “dental school” OR “dental schools” OR “Pharmacy school” OR “Pharmacy schools” OR physiotherapist OR physiotherapists OR “Physiotherapy education” OR “Physiotherapy students” OR “Physiotherapy student” OR “Physiotherapy school” OR “Physiotherapy schools” OR “Physical therapy faculty” OR “Physiotherapy faculty” OR “Physical therapy student” OR “Physical therapy students” OR “Physical therapy education” OR “Physical therapy educations” OR “Physical therapy school” OR “Physical therapy schools” OR “medical curricula” OR “medical curriculum” OR “medical faculty” OR “Medical learners” OR interns OR intern OR internship OR internships OR “medical postgraduates” OR “medical postgraduate” OR “medical graduate” OR “medical graduates” OR “medical undergraduate” OR “medical undergraduates” OR “medical student” OR “medical students” OR “medical education” OR “medical educations” OR Clerkship* OR residents OR residency OR “teaching round” OR “teaching rounds” OR “Health Personnel” OR “medical school” OR “medical schools” OR “Medical college” OR “medical universities” OR “medical university” OR “Physical therapists” OR “Physical therapist” OR physician* OR pharmacist* OR nurse* OR “medical staff” OR “health educators” OR “nursing faculty” OR “dental faculty” OR dentist* OR “pharmacy faculty” OR “health personnel” OR “allied health personnel” OR “premedical student” OR “premedical students” OR “pharmacy students” OR “pharmacy student” OR “dental student” OR “dental students” OR “nursing students” OR “Nursing education “OR “Nursing educations “OR “nursing student” OR “pharmacy graduates” OR “pharmacy undergraduates” OR “pharmacy graduate” OR “pharmacy undergraduate” OR “pharmacy postgraduates” OR “pharmacy postgraduate” OR “nursing graduates” OR “nursing graduate” OR “nursing undergraduates” OR “nursing undergraduates” OR “dental graduates” OR “dental graduate” OR “dental undergraduates” OR “dental undergraduate” OR “dental postgraduates” OR “dental postgraduate”) AND (Remediation* OR remedial*) AND (communicat* OR “interpersonal relation” OR “Interpersonal relationships” OR “Interpersonal relationship” OR “interpersonal relations” OR “Interpersonal Interaction” OR “Interpersonal Interactions”))	**191**	▪ All terms searched in the field “Topic” (which includes the fields: “Abstract”, “Title”, Author Keywords and “Keyword Plus”). ▪ Title and abstract search only is not available. No thesaurus available. No filters or limitations applied
**Psych- Info** (EBSCO) **Search Date:** 2018-06-08 **Coverage:** 1964-	TI/AB ((“psychiatrists” OR “psychiatrist” OR “premedical education” OR “dental education” OR “dental educations” OR “pharmacy education” OR “pharmacy educations” OR “nursing school” OR “nursing schools” OR “dental school” OR “dental schools” OR “pharmacy school” OR “pharmacy schools” OR “physiotherapist” OR “physiotherapists” OR “physiotherapy education” OR “physiotherapy students” OR “physiotherapy student” OR “physiotherapy school” OR “physiotherapy schools” OR “physical therapy faculty” OR “physiotherapy faculty” OR “physical therapy student” OR “physical therapy students” OR “physical therapy education” OR “physical therapy educations” OR “physical therapy school” OR “physical therapy schools” OR “medical curricula” OR “medical curriculum” OR “medical faculty” OR “medical learners” OR interns OR intern OR internship OR internships OR “medical postgraduates” OR “medical postgraduate” OR “medical graduate” OR “medical graduates” OR “medical undergraduate” OR “medical undergraduates” OR “medical student” OR “medical students” OR “medical education” OR “medical educations” OR clerkship* OR residents OR residency OR “teaching round” OR “teaching rounds” OR “health personnel” OR “medical school” OR “medical schools” OR “medical college” OR “medical universities” OR “medical university” OR “physical therapists” OR “physical therapist” OR “physiotherapist” OR “physiotherapists” OR physician* OR pharmacist* OR nurse* OR “medical staff” OR “health educators” OR “nursing faculty” OR “dental faculty” OR dentist* OR “pharmacy faculty” OR “health personnel” OR “allied health personnel” OR “premedical student” OR “premedical students” OR “pharmacy students” OR “pharmacy student” OR “dental student” OR “dental students” OR “nursing students” OR “nursing education “OR “nursing educations “OR “nursing student” OR “pharmacy graduates” OR “pharmacy undergraduates” OR “pharmacy graduate” OR “pharmacy undergraduate” OR “pharmacy postgraduates” OR “pharmacy postgraduate” OR “nursing graduates” OR “nursing graduate” OR “nursing undergraduates” OR “nursing undergraduates” OR “dental graduates” OR “dental graduate” OR “dental undergraduates” OR “dental undergraduate” OR “dental postgraduates” OR “dental postgraduate”) OR “pharmaceutical education” OR “pharmaceutical education” OR “pharmaceutical school” OR “pharmaceutical schools” OR “pharmaceutical student” OR “pharmaceutical students”) OR DE (“Dental Students” OR “Dental Education” OR “Medical Students” OR “Medical Education” OR “Medical Internship” OR “Medical Residency” OR “Nursing Education” OR “Nursing Students” OR “Allied Health Personnel” OR “Health Personnel” OR “Physical Therapists” OR “Dentists” OR “Nurses” OR “Pharmacists” OR “Physical Therapists” OR “Physicians” OR “Psychiatric Hospital Staff” OR “Clinicians” OR “Mental Health Personnel”) AND TI/AB (communicat* OR “interpersonal relation” OR “Interpersonal relationships” OR “Interpersonal relationship” OR “interpersonal relations”) OR DE (“Communication” OR “Verbal Communication” OR “Persuasive Communication” OR “Communication Skills” OR “Oral Communication” OR “Nonverbal Communication” OR “Interpersonal Communication” OR “Communication Barriers”) AND TI/AB (remediation* OR remedial*) OR DE (“Remedial Education”))	**50**	All terms searched in the fields “Title and “Abstract” (here marked as TI/AB) and in the thesaurus (DE), when available. No filters or limitations applied
**CINAHL** (EBSCO) **Search Date:** 2018-06-08 **Coverage:** 1981-	TI/AB ((“psychiatrists” OR “psychiatrist” OR “premedical education” OR “dental education” OR “dental educations” OR “pharmacy education” OR “pharmacy educations” OR “nursing school” OR “nursing schools” OR “dental school” OR “dental schools” OR “pharmacy school” OR “pharmacy schools” OR “physiotherapist” OR “physiotherapists” OR “physiotherapy education” OR “physiotherapy students” OR “physiotherapy student” OR “physiotherapy school” OR “physiotherapy schools” OR “physical therapy faculty” OR “physiotherapy faculty” OR “physical therapy student” OR “physical therapy students” OR “physical therapy education” OR “physical therapy educations” OR “physical therapy school” OR “physical therapy schools” OR “medical curricula” OR “medical curriculum” OR “medical faculty” OR “medical learners” OR interns OR intern OR internship OR internships OR “medical postgraduates” OR “medical postgraduate” OR “medical graduate” OR “medical graduates” OR “medical undergraduate” OR “medical undergraduates” OR “medical student” OR “medical students” OR “medical education” OR “medical educations” OR clerkship* OR residents OR residency OR “teaching round” OR “teaching rounds” OR “health Personnel” OR “medical school” OR “medical schools” OR “medical college” OR “medical universities” OR “medical university” OR “physical therapists” OR “physical therapist” OR “physiotherapist” OR “physiotherapists” OR physician* OR pharmacist* OR nurse* OR “medical staff” OR “health educators” OR “nursing faculty” OR “dental faculty” OR dentist* OR “pharmacy faculty” OR “health personnel” OR “allied health personnel” OR “premedical student” OR “premedical students” OR “pharmacy students” OR “pharmacy student” OR “dental student” OR “dental students” OR “nursing students” OR “nursing education “OR “nursing educations “OR “nursing student” OR “pharmacy graduates” OR “pharmacy undergraduates” OR “pharmacy graduate” OR “pharmacy undergraduate” OR “pharmacy postgraduates” OR “pharmacy postgraduate” OR “nursing graduates” OR “nursing graduate” OR “nursing undergraduates” OR “nursing undergraduates” OR “dental graduates” OR “dental graduate” OR “dental undergraduates” OR “dental undergraduate” OR “dental postgraduates” OR “dental postgraduate” OR “pharmaceutical education” OR “pharmaceutical education” OR “pharmaceutical school” OR “pharmaceutical schools” OR “pharmaceutical student” OR “pharmaceutical students”) OR MH (“Health Personnel” OR “Allied Health Personnel” OR “Dentists” OR “Nurses” OR “Physicians” “Pharmacists” OR “Faculty, Dental” OR “Education, Medical” OR “Education, Medical, Continuing” OR “Schools, Medical” OR “Internship and Residency” OR “Interns and Residents” OR “Students, Medical” OR “Students, Dental” OR “Students, Pharmacy” OR “Faculty, Medical” OR “Faculty, Nursing” OR “Medical Staff” OR “Pharmacists” OR “Physical Therapists” OR “Schools, Dental” OR “Education, Dental” OR “Psychiatrists” OR “Education, Nursing” OR “Education, Nursing, Diploma Programs” OR “Education, Nursing, Practical” OR “Education, Nursing, Masters” OR “Education, Nursing, Theory-Based” OR “Education, Nursing, Research-Based” OR “Education, Nursing, Graduate” OR “Education, Nursing, Continuing” OR “Education, Nursing, Baccalaureate” OR “Students, Nursing, Male” OR “Students, Nursing, Graduate” OR “Students, Nursing, Masters” OR “Students, Nursing, Diploma Programs” OR “Students, Nursing, Baccalaureate” OR “Students, Nursing, Practical” OR “Schools, Nursing” OR “Education, Nursing, Diploma Programs” OR “School Health Nursing” OR “Education, Pharmacy” OR “Education, Premedical”) AND TI/AB (communicat* OR “interpersonal relation” OR “interpersonal relationships” OR “interpersonal relationship” OR “interpersonal relations” OR “interpersonal Interaction” OR “interpersonal interactions”) OR MH (“Interpersonal Relations” OR “Communication” OR “Nonverbal Communication” OR “Communication Skills Training” OR “Communication Skills” OR “Communication Barriers” OR “Persuasive Communication”) AND TI/AB (remediation* OR remedial*) OR MH (“Remedial Teaching”))	**51**	All terms searched in the fields “Title” (“.ti”), “Abstract” (“.ab”) and in the “Thesaurus” (“/”) when available. No filters or limitations applied
**EMBASE** (OVID) **Search Date:** 2018-06-10 **Coverage:** 1974-	TI/AB ((“psychiatrists” OR “psychiatrist” OR “premedical education” OR “dental education” OR “dental educations” OR “pharmacy education” OR “pharmacy educations” OR “nursing school” OR “nursing schools” OR “dental school” OR “dental schools” OR “pharmacy school” OR “pharmacy schools” OR “physiotherapist” OR “physiotherapists” OR “physiotherapy education” OR “physiotherapy students” OR “physiotherapy student” OR “physiotherapy school” OR “physiotherapy schools” OR “physical therapy faculty” OR “physiotherapy faculty” OR “physical therapy student” OR “physical therapy students” OR “physical therapy education” OR “physical therapy educations” OR “physical therapy school” OR “physical therapy schools” OR “medical curricula” OR “medical curriculum” OR “medical faculty” OR “medical learners” OR interns OR intern OR internship OR internships OR “medical postgraduates” OR “medical postgraduate” OR “medical graduate” OR “medical graduates” OR “medical undergraduate” OR “medical undergraduates” OR “medical student” OR “medical students” OR “medical education” OR “medical educations” OR clerkship* OR residents OR residency OR “teaching round” OR “teaching rounds” OR “health Personnel” OR “medical school” OR “medical schools” OR “medical college” OR “medical universities” OR “medical university” OR “physical therapists” OR “physical therapist” OR “physiotherapist” OR “physiotherapists” OR physician* OR pharmacist* OR nurse* OR “medical staff” OR “health educators” OR “nursing faculty” OR “dental faculty” OR dentist* OR “pharmacy faculty” OR “health personnel” OR “allied health personnel” OR “premedical student” OR “premedical students” OR “pharmacy students” OR “pharmacy student” OR “dental student” OR “dental students” OR “nursing students” OR “nursing education “OR “nursing educations “OR “nursing student” OR “pharmacy graduates” OR “pharmacy undergraduates” OR “pharmacy graduate” OR “pharmacy undergraduate” OR “pharmacy postgraduates” OR “pharmacy postgraduate” OR “nursing graduates” OR “nursing graduate” OR “nursing undergraduates” OR “nursing undergraduates” OR “dental graduates” OR “dental graduate” OR “dental undergraduates” OR “dental undergraduate” OR “dental postgraduates” OR “dental postgraduate” OR “pharmaceutical education” OR “pharmaceutical education” OR “pharmaceutical school” OR “pharmaceutical schools” OR “pharmaceutical student” OR “pharmaceutical students”) OR (dental education/ OR dentist/ OR dental student/OR nursing education/ OR nursing student/ OR baccalaureate nursing student/ OR graduate nursing student/ OR male nursing student/ OR nurse/ OR pharmacy student/ OR pharmacist/ OR medical student/ OR medical education/ OR medical school/ OR physician/ OR residency education/ OR clinical education/ OR health care personnel/OR physiotherapist/ OR psychiatrist/) AND TI/AB (communicat* OR “interpersonal relation” OR “Interpersonal relationships” OR “interpersonal relationship” OR “interpersonal relations” OR “interpersonal Interaction” OR “interpersonal interactions”) OR (interpersonal communication/ OR communication skill/ OR nonverbal communication/ OR verbal communication**/** OR persuasive communication/ OR interdisciplinary communication/) AND TI/AB (remediation* OR remedial*))	**185**	All terms searched in the fields “Title” (“.ti”), “Abstract” (“.ab”) and in the “Thesaurus” (“/”) when available. No filters or limitations applied “Remediation” not included in the thesaurus. The thesaurus term: “communication disorder” included in the search here as it refers to “communication barriers” etc.
**ERIC** (OVID) **Search Date:** 2018-06-07 **Coverage:** 1965-	TI/AB (“psychiatrists” OR “psychiatrist” OR “premedical education” OR “dental education” OR “dental educations” OR “pharmacy education” OR “pharmacy educations” OR “nursing school” OR “nursing schools” OR “dental school” OR “dental schools” OR “pharmacy school” OR “pharmacy schools” OR “physiotherapist” OR “physiotherapists” OR “physiotherapy education” OR “physiotherapy students” OR “physiotherapy student” OR “physiotherapy school” OR “physiotherapy schools” OR “physical therapy faculty” OR “physiotherapy faculty” OR “physical therapy student” OR “physical therapy students” OR “physical therapy education” OR “physical therapy educations” OR “physical therapy school” OR “physical therapy schools” OR “medical curricula” OR “medical curriculum” OR “medical faculty” OR “medical learners” OR interns OR intern OR internship OR internships OR “medical postgraduates” OR “medical postgraduate” OR “medical graduate” OR “medical graduates” OR “medical undergraduate” OR “medical undergraduates” OR “medical student” OR “medical students” OR “medical education” OR “medical educations” OR clerkship* OR residents OR residency OR “teaching round” OR “teaching rounds” OR “health Personnel” OR “medical school” OR “medical schools” OR “medical college” OR “medical universities” OR “medical university” OR “physical therapists” OR “physical therapist” OR “physiotherapist” OR “physiotherapists” OR physician* OR pharmacist* OR nurse* OR “medical staff” OR “health educators” OR “nursing faculty” OR “dental faculty” OR dentist* OR “pharmacy faculty” OR “health personnel” OR “allied health personnel” OR “premedical student” OR “premedical students” OR “pharmacy students” OR “pharmacy student” OR “dental student” OR “dental students” OR “nursing students” OR “nursing education “OR “nursing educations “OR “nursing student” OR “pharmacy graduates” OR “pharmacy undergraduates” OR “pharmacy graduate” OR “pharmacy undergraduate” OR “pharmacy postgraduates” OR “pharmacy postgraduate” OR “nursing graduates” OR “nursing graduate” OR “nursing undergraduates” OR “nursing undergraduates” OR “dental graduates” OR “dental graduate” OR “dental undergraduates” OR “dental undergraduate” OR “dental postgraduates” OR “dental postgraduate” OR “pharmaceutical education” OR “pharmaceutical education” OR “pharmaceutical school” OR “pharmaceutical schools” OR “pharmaceutical student” OR “pharmaceutical students”) OR (dental schools/ OR dentistry/ OR nursing students/ OR allied health occupations education/ OR nurses/ OR nursing education/ OR medical schools/ OR graduate medical education/ OR medical education/ OR medical students/ OR medical schools/ OR graduate medical education/ OR medical education/ OR medical school faculty/ OR medical students/ OR pharmacy/ OR pharmaceutical education/ OR premedical students/ OR undergraduate students/ OR medical education/ OR medical students/ OR nursing education/ OR clinical experience/ OR health personnel/ OR allied health personnel/ OR nurses/ OR physicians/ OR psychologists/ OR nursing education/ OR pharmaceutical education/) AND TI/AB (communicat* OR “interpersonal relation” OR “Interpersonal relationships” OR “interpersonal relationship” OR “interpersonal relations” OR “interpersonal Interaction” OR “interpersonal interactions”) OR (verbal communication/ OR nonverbal communication/ OR interpersonal relationship/) AND TI/AB (remediation* OR remedial*) OR (remedial instruction/))	**46**	All terms searched in the fields “Title” (“.ti”), “Abstract” (“.ab”) and in the “Thesaurus” (“/”) when available. No filters or limitations applied
**Total numbers of references**			**988**
**Duplicates removed**			**486**
**Total numbers of references after de-duplication**			**502**

**Table 5:** Updated search in PubMed and scopus, 2019-05-14

Source and search date	Search String	Result	Notes
**PubMed** **Search Date:** 2019-05-14	(((“psychiatrists”[Title/Abstract] OR “psychiatrist”[Title/Abstract] OR “premedical education”[Title/Abstract] OR “dental education”[Title/Abstract] OR “dental educations” [Title/Abstract] OR “pharmaceutical education”[Title/Abstract] OR “pharmaceutical education” [Title/Abstract] OR “pharmaceutical school” [Title/Abstract] OR “pharmaceutical schools” [Title/Abstract] OR “pharmaceutical student” [Title/Abstract] OR “pharmaceutical students” [Title/Abstract] OR “pharmacy education”[Title/Abstract] OR “pharmacy educations”[Title/Abstract] OR “nursing school”[Title/Abstract] OR “nursing schools”[Title/Abstract] OR “dental school”[Title/Abstract] OR “dental schools”[Title/Abstract] OR (“pharmacy school”[Title/Abstract] OR “pharmacy schools”[Title/Abstract] OR physiotherapist [Title/Abstract] OR physiotherapists [Title/Abstract] OR “physiotherapy education”[Title/Abstract] OR “physiotherapy students”[Title/Abstract] OR “physiotherapy student”[Title/Abstract] OR “physiotherapy school”[Title/Abstract] OR “physiotherapy schools”[Title/Abstract] OR “physical therapy faculty”[Title/Abstract] OR “physiotherapy faculty”[Title/Abstract] OR “physical therapy student”[Title/Abstract] OR “physical therapy students”[Title/Abstract] OR “physical therapy education”[Title/Abstract] OR “physical therapy educations”[Title/Abstract] OR “physical therapy school”[Title/Abstract] OR “physical therapy schools”[Title/Abstract] OR “Schools, Dental”[Mesh] OR “Schools, Nursing”[Mesh] OR “Schools, Pharmacy”[Mesh] OR “Education, Pharmacy”[Mesh] OR “Education, Premedical”[Mesh] OR “Education, Dental”[Mesh] OR “Education, Nursing”[Mesh] OR “medical curricula”[Title/Abstract] OR “medical curriculum”[Title/Abstract] OR “medical faculty”[Title/Abstract] OR “medical learners”[Title/Abstract] OR interns [Title/Abstract] OR intern [Title/Abstract] OR internship [Title/Abstract] OR internships [Title/Abstract] OR “Education, Medical”[Mesh] OR “Internship and Residency”[Mesh] OR “Clinical Clerkship”[Mesh] OR “Students, Medical”[Mesh] OR “medical postgraduates”[Title/Abstract] OR “medical postgraduate”[Title/Abstract] OR “medical graduate”[Title/Abstract] OR “medical graduates”[Title/Abstract] OR “medical undergraduate”[Title/Abstract] OR “medical undergraduates”[Title/Abstract] OR “medical student”[Title/Abstract] OR “medical students”[Title/Abstract] OR “medical education”[Title/Abstract] OR “medical educations”[Title/Abstract] OR clerkship*[Title/Abstract] OR resident [Title/Abstract] OR residents [Title/Abstract] OR residency [Title/Abstract] OR “teaching round”[Title/Abstract] OR “teaching rounds”[Title/Abstract] OR “Health Personnel”[Mesh] OR “medical school”[Title/Abstract] OR “medical schools”[Title/Abstract] OR “medical college”[Title/Abstract] OR “medical universities”[Title/Abstract] OR “medical university”[Title/Abstract] OR “Schools, Medical”[Mesh] OR “physical therapists” [Title/Abstract] OR “physical therapist” [Title/Abstract] OR physician*[Title/Abstract] OR pharmacist*[Title/Abstract] OR nurse*[Title/Abstract] OR “medical staff”[Title/Abstract] OR “health educators”[Title/Abstract] OR “nursing faculty”[Title/Abstract] OR “dental faculty”[Title/Abstract] OR dentist*[Title/Abstract] OR “pharmacy faculty”[Title/Abstract] OR “health personnel”[Title/Abstract] OR “allied health personnel”[Title/Abstract] OR “Faculty, Nursing”[Mesh] OR “Faculty, Medical”[Mesh] OR “Faculty, Dental”[Mesh] OR “Students, Nursing”[Mesh] OR “Students, Pharmacy”[Mesh] OR “Students, Dental”[Mesh] OR “Students, Premedical”[Mesh] OR “premedical student”[Title/Abstract] OR “premedical students”[Title/Abstract] OR “pharmacy students”[Title/Abstract] OR “pharmacy student”[Title/Abstract] OR “dental student”[Title/Abstract] OR “dental students”[Title/Abstract] OR “nursing students”[Title/Abstract] OR “Nursing education“[Title/Abstract] OR “Nursing educations“[Title/Abstract] OR “nursing student”[Title/Abstract] OR “pharmacy graduates”[Title/Abstract] OR “pharmacy undergraduates”[Title/Abstract] OR “pharmacy graduate”[Title/Abstract] OR “pharmacy undergraduate”[Title/Abstract] OR “pharmacy postgraduates”[Title/Abstract] OR “pharmacy postgraduate”[Title/Abstract] OR “nursing graduates”[Title/Abstract] OR “nursing graduate”[Title/Abstract] OR “nursing undergraduates”[Title/Abstract] OR “nursing undergraduates”[Title/Abstract] OR “dental graduates”[Title/Abstract] OR “dental graduate”[Title/Abstract] OR “dental undergraduates”[Title/Abstract] OR “dental undergraduate”[Title/Abstract] OR “dental postgraduates”[Title/Abstract] OR “dental postgraduate”[Title/Abstract]) AND (“Remedial Teaching”[Mesh] OR Remediation*[Title/Abstract] OR remedial* [Title/Abstract]) AND (“Teach-Back Communication”[Mesh] OR “Health Communication”[Mesh] OR “Interdisciplinary Communication”[Mesh] OR “Persuasive Communication”[Mesh] OR “Nonverbal Communication”[Mesh] OR “Communication Barriers”[Mesh] OR “communication”[Mesh] OR communicat*[Title/Abstract] OR “interpersonal relation”[Title/Abstract] OR “interpersonal relationships” [Title/Abstract] OR “interpersonal relationship” [Title/Abstract] OR “interpersonal relations”[Title/Abstract] OR “interpersonal Interaction” [Title/Abstract] OR “interpersonal Interactions” [Title/Abstract] OR “Interpersonal Relations”[Mesh])))	**12**	All terms searched in field “Title/Abstract” and in “MeSH” when available. Filter for publication year, 2018-06-08- 2019-05-14 applied No duplicates detected within the result
**Scopus** **Search Date:** 2019-05-14	(TITLE-ABS-KEY (“psychiatrists” OR “psychiatrist” OR “premedical education” OR “dental education” OR “dental educations” OR “pharmaceutical education” OR “pharmaceutical education” OR “pharmaceutical school” OR “pharmaceutical schools” OR “pharmaceutical student” OR “pharmaceutical students” OR “pharmacy educations” OR “nursing school” OR “nursing schools” OR “dental school” OR “dental schools” OR “pharmacy school” OR “pharmacy schools” OR physiotherapist OR physiotherapists OR “physiotherapy education” OR “physiotherapy students” OR “physiotherapy student” OR “physiotherapy school” OR “physiotherapy schools” OR “physical therapy faculty” OR “physiotherapy faculty” OR “physical therapy student” OR “physical therapy students” OR “physical therapy education” OR “physical therapy educations” OR “physical therapy school” OR “physical therapy schools” OR “medical curricula” OR “medical curriculum” OR “medical faculty” OR “medical learners” OR interns OR intern OR internship OR internships OR “medical postgraduates” OR “medical postgraduate” OR “medical graduate” OR “medical graduates” OR “medical undergraduate” OR “medical undergraduates” OR “medical student” OR “medical students” OR “medical education” OR “medical educations” OR clerkship* OR residents OR residency OR “teaching round” OR “teaching rounds” OR “health Personnel” OR “medical school” OR “medical schools” OR “Medical college” OR “medical universities” OR “medical university” OR “physical therapists” OR “physical therapist” OR physician* OR pharmacist* OR nurse* OR “medical staff” OR “health educators” OR “nursing faculty” OR “dental faculty” OR dentist* OR “pharmacy faculty” OR “health personnel” OR “allied health personnel” OR “premedical student” OR “premedical students” OR “pharmacy students” OR “pharmacy student” OR “dental student” OR “dental students” OR “nursing students” OR “nursing education” OR “nursing educations” OR “nursing student” OR “pharmacy graduates” OR “pharmacy undergraduates” OR “pharmacy graduate” OR “pharmacy undergraduate” OR “pharmacy postgraduates” OR “pharmacy postgraduate” OR “nursing graduates” OR “nursing graduate” OR “nursing undergraduates” OR “nursing undergraduates” OR “dental graduates” OR “dental graduate” OR “dental undergraduates” OR “dental undergraduate” OR “dental postgraduates” OR “dental postgraduate”) AND (TITLE-ABS-KEY (communicat* OR “interpersonal relation” OR “interpersonal relationships” OR “interpersonal relationship” OR “interpersonal relations” OR “interpersonal interaction” OR “interpersonal interactions”) AND (TITLE-ABS-KEY (remediation* OR remedial*))	**20**	All selected search terms searched in the fields for “Title”, Abstract” and “Keywords”, here marked with “TI-ABS-KEY.” No thesaurus available Filter for publication year, 2018- the search date were applied. The references form 2018 were screened by hand to only include studies published after 2018-06-08- in the result. No duplicates detected within the result
**Total numbers of references**			**32**
**Duplicates removed**			**10**
**Total numbers of references after de-duplication**			**22**

**Table 6:** Grey sources

Source and search date	Search string	Result	Notes
**ProQuest Dissertation and Theses** **Search date:** 2019-05-18	(((“psychiatrists” OR “psychiatrist” OR “premedical education” OR “dental education” OR “dental educations” OR “pharmaceutical” OR “pharmacy educations” OR “nursing school” OR “nursing schools” OR “dental school” OR “dental schools” OR “pharmacy school” OR “pharmacy schools” OR physiotherapist OR physiotherapists OR “physiotherapy education” OR “physiotherapy students” OR “physiotherapy student” OR “physiotherapy school” OR “physiotherapy schools” OR “physical therapy faculty” OR “physiotherapy faculty” OR “physical therapy student” OR “physical therapy students” OR “physical therapy education” OR “physical therapy educations” OR “physical therapy school” OR “physical therapy schools” OR “medical curricula” OR “medical curriculum” OR “medical faculty” OR “medical learners” OR interns OR intern OR internship OR internships OR “medical postgraduates” OR “medical postgraduate” OR “medical graduate” OR “medical graduates” OR “medical undergraduate” OR “medical undergraduates” OR “medical student” OR “medical students” OR “medical education” OR “medical educations” OR clerkship* OR residents OR residency OR “teaching round” OR “teaching rounds” OR “health personnel” OR “medical school” OR “medical schools” OR “medical college” OR “medical universities” OR “medical university” OR “physical therapists” OR “physical therapist” OR physician* OR pharmacist* OR nurse* OR “medical staff” OR “health educators” OR “nursing faculty” OR “dental faculty” OR dentist* OR “pharmacy faculty” OR “health personnel” OR “allied health personnel” OR “premedical student” OR “premedical students” OR “pharmacy students” OR “pharmacy student” OR “dental student” OR “dental students” OR “nursing students” OR “nursing education” OR “nursing educations” OR “nursing student” OR “pharmacy graduates” OR “pharmacy undergraduates” OR “pharmacy graduate” OR “pharmacy undergraduate” OR “pharmacy postgraduates” OR “pharmacy postgraduate” OR “nursing graduates” OR “nursing graduate” OR “nursing undergraduates” OR “nursing undergraduates” OR “dental graduates” OR “dental graduate” OR “dental undergraduates” OR “dental undergraduate” OR “dental postgraduates” OR “dental postgraduate”) AND (remediation* OR remedial*) AND (communicat* OR “interpersonal relation” OR “interpersonal relationships” OR “interpersonal relationship” OR “interpersonal relations” OR “interpersonal Interaction” OR “interpersonal interactions”)))	34	Search in “All fields Except Full text”. No filters or limitations applied
**Ethos** **Search date:** 2019-05-18	remediation AND communication AND student	12	* The search function in this source is very limited. A broad search was conducted. No filters or limitations applied.
	remediation AND communication AND medical	9	
	remediation AND communication AND physicians	1	
	remediation AND interpersonal AND medical	1	
	remediation AND interpersonal	9	
**Open Grey** (Grey literature in Europe) **Search date:** 2019-05-18	remediation AND communication	14	* The search function in this source is very limited. A broad search was conducted.
	remediation AND communication AND students	2	
	remediation AND communication AND medical	1	
	remediation AND interpersonal AND medical	0	
	remediation AND interpersonal	0	
	remediation AND communication AND physicians	0	
**BASE** (Bielefeld Academic Search Engine) **Search date:** 2019-05-18	tit:communicat* tit:remedia*	70	Searched in the field for “Title”
	subj:remedia* subj:communica*	181	Searched in the field for “Subject”
	subj:remedia* subj:communica* subj:medical*	1	
	subj:interpersonal* subj:remedia* subj:medical*	1	
	subj:interpersonal* subj:remedia* subj:student*	2	
**The New York Academy of Grey Literature Reports** **Search date:** 2019-05-18	remediation AND communication	1	
	remediation AND interpersonal	0	
	remediation AND students	0	
**British Library Main Catalogue** **Search date:** 2019-05-18	remedia* AND communica*	5	Searched with publication limitation: “Theses”
	remedia* AND communica* AND medical*	0	
	remedia* AND communica* AND medical*	81	Searched in “Whole Catalogue”
	remedia* AND interpersonal* AND medical*	10	
	remedia* AND interpersonal*	104	
	remedia* AND interpersonal* AND student*	11	
**WorldCat library catalogue** **Search date:** 2019-05-18	ti:remedia* ti:communicat* ti:medical*	21	Searched in the field for “Title” Searched in the field for “Title”
	ti:remedia* ti:interpersonal*	43	
**Total numbers of references**			**614**
**Duplicates removed**			**207**
**Total numbers of references after de-duplication**			**407**

## Abbreviations

ACGME: Accreditation Council for Graduate Medical Education
ABMS: American Board of Medical Specialties
BOE-PG: Board of Examiners for Postgraduate Programs
CCS: Communication and Consultation Skills
CPX: Clinical Performance Examination
CE: Certifying Exam
CME: Continuing Medical Education
CS: Clinical Skills
ECFMG: Educational Commission for Foreign Medical Graduates
FSMB: Federation of State Medical Boards
IEP: Individualized Education Plan
IRRC: Internal Residency Review Committee
MERSQI: Medical Education Research Study Quality Instrument
NBME: National Board of Medical Examiner
OSCE: Objective Structured Clinical Examination
PD: Program Director
PDI: Patient-Doctor Interaction
PIC: Professional Inspection Committee
SOI: Structured Oral Interview
SP: Standardized Patient
SPE: Standardized Patient Educator
